# The Wake Forest IASDR: A Resource for Studying the Effects of Caloric Restriction on Health Outcomes in Older Adults

**DOI:** 10.1093/geroni/igab046.298

**Published:** 2021-12-17

**Authors:** Denise Houston, Jamie Justice, Anne Newman

## Abstract

Over the past 25+ years, a focus of the Wake Forest Claude D. Pepper Older Americans Independence Center (OAIC) has been to study the consequences of and treatments for geriatric obesity. The Wake Forest OAIC has provided support for 18 clinical trials of caloric restriction (CR), with and without various exercise regimens, in 2,545 adults (71% women, 21% African American) with a mean±SD age of 67.5±5.9 years and BMI ≥27 kg/m2. A priority of the Wake Forest OAIC is to collate and store common data (e.g., demographics, physical performance, cognitive function), biospecimens (blood, muscle, adipose), and images (DXA, CT) from these trials in the Integrated Aging Studies Databank and Repository (IASDR; https://www.peppercenter.org/public/dspIASDR.cfm). This IASDR serves as a resource for the scientific community to foster new scientific questions and analyses. This symposium will provide an overview of CR trials and participants included in the IASDR and how the IASDR supports secondary analyses of CR by highlighting several secondary analyses using data and/or samples from the IASDR. Justice and colleagues examined the effect of CR on a geroscience-guided biomarker index using blood samples from the biorepository. Weaver and colleagues examined the effect of different exercise regimens on CT-derived muscle and bone measures during CR. Miller and colleagues pooled data from 11 trials to determine if CR-induced appendicular lean mass loss is associated with changes in physical performance. Finally, Hsieh and colleagues pooled data from eight trials to examine whether the effect of CR on gait speed differed by baseline BMI and inflammation.

